# Cervical Magnetic Resonance Imaging Profiles and Their Association with Cervical Pain in Dentists: A Cluster Analysis Study

**DOI:** 10.3390/jcm15020536

**Published:** 2026-01-09

**Authors:** Ana López-Morales, Aitor Baño-Alcaraz, Manuel López-Nicolás, José Antonio García-Vidal, Germán Cánovas-Ambit

**Affiliations:** 1 Department of Dermatology, Stomatology, Radiology and Physical Medicine, University of Murcia, 30008 Murcia, Spain; lopezmoralesana0@gmail.com (A.L.-M.); malopez@um.es (M.L.-N.); 2 Department of Physiotherapy, Faculty of Medicine, University of Murcia, Campus de Espinardo, 30100 Murcia, Spain; aitor.bano@um.es (A.B.-A.); german.canovas@um.es (G.C.-A.); 3 Biomedical Research Institute of Murcia IMIB-Pascual Parrilla, Campus de Espinardo, 30100 Murcia, Spain

**Keywords:** neck pain, dentists, cervical MRI, degenerative changes, cluster analysis, musculoskeletal disorders, occupational health

## Abstract

**Background/Objectives:** Neck pain is highly prevalent among dentists and has been linked to occupational exposure and cervical degeneration. However, the relationship between cervical MRI findings and symptoms remains inconsistent. This study aimed to explore MRI-based cervical structural profiles in active dentists and examine their associations with neck pain, disability, and participant characteristics. **Methods:** A cross-sectional study was conducted in 57 practicing dentists. Participants reported neck pain and completed the Numeric Pain Rating Scale and the Neck Disability Index (NDI). Cervical MRI scans were assessed by an experienced musculoskeletal radiologist. An exploratory hierarchical cluster analysis (complete linkage, Euclidean distance) was applied using MRI degenerative variables to identify structural profiles, followed by bivariate comparisons with clinical and occupational factors. **Results:** Degenerative MRI findings were common (disc bulging, 66.7%; disc herniation, 54.4%). Two MRI-based profiles were identified, one characterized by a higher burden of degenerative findings (including disc and facet changes) (70.2%), and another with fewer/milder degenerative features (29.8%). Neck pain and NDI scores ≥ 20 were more frequent in the higher-degeneration profile (*p* = 0.001 and *p* = 0.004, respectively). Age showed a non-linear pattern, with younger dentists reporting pain despite milder MRI changes, whereas older dentists showed more degeneration with fewer symptoms. **Conclusions:** In this exploratory study, individual MRI findings were not independently associated with neck pain, while a higher overall burden of degenerative changes tended to co-occur with greater symptom reporting and disability. These findings should be interpreted as hypothesis-generating and warrant confirmation in larger, longitudinal studies.

## 1. Introduction

Neck pain (NP) is among the most prevalent musculoskeletal disorders globally and constitutes a leading cause of work-related disability, with lifetime prevalence rates estimated between 50% and 70% in the general population [1,2]. Within healthcare occupations, cervical spine disorders are particularly frequent among professionals who perform prolonged manual tasks or maintain static postures involving sustained cervical flexion [3]. Among these, dentists represent a high-risk occupational group, with reported prevalence rates of NP ranging from 60% to 95% across studies [4,5,6]. The occurrence of NP in this population has been linked to a multifactorial etiology involving demographic factors (such as age and years of professional experience), occupational factors (including working hours, prolonged postural maintenance, and the use of ergonomic supports), and structural factors (cervical spine degenerative changes), all of which may contribute to symptom development and persistence [5,7,8,9].

Magnetic resonance imaging (MRI) is considered the reference technique for the in vivo evaluation of structural and degenerative changes in the cervical spine [10,11,12,13]. Although MRI provides detailed morphological information, the scientific literature reports inconsistent findings regarding its association with NP. Numerous asymptomatic individuals present degenerative changes comparable to those observed in symptomatic patients, suggesting that the mere presence of structural abnormalities does not necessarily correspond to clinical manifestations [14,15,16]. In the case of dentists, the available evidence on cervical MRI findings remains limited and has not conclusively established whether specific structural alterations are directly associated with neck pain [17].

The lack of correspondence between radiological findings and clinical presentation, a phenomenon also documented in other medical domains [18,19,20], indicates that structural degeneration alone is insufficient to explain symptom expression. Therefore, more integrative analytical frameworks are required to simultaneously account for morphological, clinical, and occupational determinants. In this context, multivariate analytical methods, such as cluster analysis, have gained increasing relevance in health research [21]. These approaches enable the simultaneous examination of multiple variables, allowing the identification of subgroups of individuals who share similar profiles, in order to explore the relevance of those profiles [21,22].

Given the inconsistent association between individual cervical MRI findings and neck pain, exploratory multivariate approaches may offer additional insight by examining patterns of co-occurring structural changes rather than isolated abnormalities typically analyzed using routine univariate methods. Cluster analysis was therefore employed as a hypothesis-generating method to identify potential MRI-based structural profiles among dentists. This approach is intended to describe morphological configurations and not to establish causal relationships. Importantly, cluster analysis has well-recognized limitations, particularly in studies with small sample sizes, and results should be interpreted with caution.

Although a biopsychosocial perspective is conceptually relevant to the understanding of neck pain, the present study focuses exclusively on structural and occupational factors. Psychosocial variables were not directly measured and are therefore not addressed analytically, representing an important limitation that should be considered when interpreting the findings.

To our knowledge, dentists’ profiles based on cervical MRI findings have not yet been investigated. The objective of this study was to identify dentists’ profiles derived from cervical MRI findings and to determine their association with the presence of NP, and with demographic and occupational risk factors for NP. Furthermore, the study aimed to examine the relationship between specific individual MRI-detected cervical alterations and NP.

## 2. Materials and Methods

### 2.1. Design and Participants

A cross-sectional observational study was conducted among active dentists registered in the Professional College of Dentists and Stomatologists of Murcia (Spain). Recruitment was performed through the College’s internal communication channels, which facilitated the dissemination of study information and voluntary participation. Participants were eligible if they were currently practicing dentistry and had at least one year of continuous professional experience. Exclusion criteria included a history of cervical spine surgery, neurological or systemic disorders, or any contraindication for undergoing magnetic resonance imaging (MRI). All participants provided written informed consent prior to enrollment.

The study was conducted in accordance with the Declaration of Helsinki and approved by the Ethics Committee of the University of Murcia (protocol code CEI-4737/2023).

This study was designed as an exploratory, cross-sectional investigation. Participants were recruited on a voluntary basis through internal communication channels of the Professional College of Dentists and Stomatologists of Murcia. As participation was voluntary, no formal response rate could be calculated, and information on non-participants was not available. Consequently, the potential for selection bias cannot be excluded. No formal sample size or statistical power calculation was performed, as the primary objective of the study was exploratory and hypothesis-generating.

### 2.2. Data Collection

Data collection was conducted in a single visit at an authorized radiology unit. During this session, demographic, occupational, and pain-related measures were obtained, and cervical magnetic resonance imaging (MRI) was performed.

#### 2.2.1. Demographic and Occupational Characteristics

Demographic and occupational variables potentially associated with MRI alterations were collected through a structured questionnaire: sex, age (categorized as <40, 40–49, and >49 years), years of professional experience, weekly working hours (<36 or ≥36 h), usual working position (sitting, standing, or alternating), and type of chair commonly used (without arm support, with one arm support, or with two arm supports).

#### 2.2.2. Pain and MRI Measures

Neck pain was assessed by asking participants whether they had experienced cervical discomfort during or after their professional activity (yes/no). Pain intensity was measured using a numeric rating scale (0–10), a widely used and validated method for pain assessment [23]. Disability was evaluated using the Neck Disability Index (NDI), with scores >15 considered clinically significant [24,25]. Pain was assessed in relation to professional activity, which may have influenced participants’ attribution of symptoms to work-related exposure.

MRI scans were obtained using a 1.5 Tesla General Electric Signa HDxt scanner (GE Healthcare, Milwaukee, WI, USA) with standard sagittal and axial T1- and T2-weighted sequences. Image acquisition focused on intervertebral discs, vertebral endplates, and facet joints. All images were evaluated by a musculoskeletal radiologist with more than five years of experience, blinded to all clinical and occupational data.

### 2.3. Statistical Analysis

Descriptive statistics were used to summarize participants’ demographic, occupational, clinical, and MRI characteristics. Categorical variables were reported as frequencies and percentages, and continuous variables as means and standard deviations. Group comparisons according to neck pain status were performed using chi-square or Fisher’s exact tests, as appropriate. To explore patterns of co-occurring cervical MRI findings, an exploratory hierarchical cluster analysis was performed. The analysis followed these steps: first, only MRI-derived degenerative variables were selected (disc bulging, disc herniation, annular fissure, canal stenosis, osteophytes, and facet joint abnormalities), all coded as binary variables (presence/absence). Second, a similarity matrix was computed using squared Euclidean distance. Third, participants were grouped using a hierarchical clustering procedure with the complete linkage method. The resulting dendrogram was visually inspected, and changes in agglomeration coefficients were examined to identify the most interpretable clustering solution. Based on this inspection, a two-cluster solution was selected as the most parsimonious and clinically interpretable grouping.

Following cluster identification, participants were assigned to clusters and compared in terms of pain status, disability, demographic, and occupational characteristics using chi-square or Fisher’s exact tests. Effect sizes (Cramér’s V) and 95% confidence intervals were calculated for key associations to provide an estimate of the magnitude of observed effects.

Given the exploratory and hypothesis-generating nature of the study, no formal adjustment for multiple comparisons was applied, and no cluster stability or validation analyses were performed. Statistical significance was set at *p* < 0.05. All analyses were conducted using SPSS Statistics for Windows, version 26 (IBM Corp., Armonk, NY, USA).

### 2.4. Data Availability

The data supporting the findings of this study are available from the corresponding author upon reasonable request. Due to privacy and ethical restrictions, the dataset is not publicly available.

### 2.5. Use of Generative Artificial Intelligence

Generative artificial intelligence (GenAI) tools were used exclusively for language editing and grammar improvement during manuscript preparation. No GenAI tools were used for study design, data collection, analysis, or interpretation.

### 2.6. Protocols and Materials

No new materials or protocols were developed for this study. All procedures followed established and validated methods as cited above.

## 3. Results

### 3.1. Participants

A total of 57 dentists were included in the study. All participants completed the full assessment with no missing data. Table 1 summarizes the demographic, occupational, pain-related, and MRI characteristics of the sample. The mean (SD) professional experience was 18.3 (SD = 12.2) years, and 54.4% of participants were women. Among MRI findings, the most prevalent structural changes were disc bulging (66.7%) and herniated disc (54.4%), followed by osteophyte formation (35.1%), facet joint abnormalities (29.8%). Overall, 70.2% of participants reported cervical pain during or after their professional activity. As shown in Table 1, except for NDI, non-statistically significant differences were observed between groups in terms of sex, age, years of professional experience, working hours, posture, chair support, or specific MRI findings. All participants were recruited from a single professional college, which may limit the generalizability of the results.

Accordingly, clusters were labelled descriptively based on the relative burden of co-occurring degenerative MRI findings and were not intended to represent clinically distinct or pain-defining phenotypes.

### 3.2. MRI-Based Clusters and Pain

As shown in Table 2, the cluster analysis identified two MRI-based structural profiles. Cluster 1 comprised 40 participants (70.2%), whereas Cluster 2 included 17 participants (29.8%). No individual MRI finding differed significantly between clusters. Rather than being defined by single abnormalities, the clusters reflected differences in the overall burden and co-occurrence of degenerative MRI findings. In an additional exploratory analysis, a cumulative degenerative burden score was calculated for each participant by summing the number of degenerative MRI findings present, yielding a possible range from 0 to 6 findings per participant. Descriptively, participants reporting neck pain showed a higher mean (SD) cumulative degenerative burden compared with asymptomatic participants (2.37 ± 1.54 vs. 1.38 ± 1.30 degenerative findings per participant, respectively). Given the exploratory nature of the study and the small size of the asymptomatic subgroup, these results are presented descriptively and should be interpreted as hypothesis-generating.

Importantly, the clustering procedure was based exclusively on MRI-derived variables, and pain and disability measures were not used to define the clusters. Therefore, any observed differences in pain or disability between clusters should be interpreted as descriptive associations rather than defining characteristics of the structural profiles. Thus, the terms used to describe the clusters reflect differences in degenerative burden rather than diagnostic or prognostic categories.

Cluster 1 showed a higher combined prevalence of degenerative changes across multiple cervical structures, including disc and facet joint alterations, whereas Cluster 2 was characterized by fewer and less extensive degenerative findings. Pain status and disability were not included in the clustering procedure and were examined only in subsequent descriptive comparisons between clusters. In these comparisons, neck pain and higher NDI scores were more frequently observed in Cluster 1.

### 3.3. Participants’ Characteristics Related to MRI-Based Clusters with and Without Pain

Table 3 presents the distribution of demographic and occupational characteristics stratified by MRI-based cluster membership and pain status. Within each MRI-based cluster, participants are further subdivided into those reporting neck pain and those without pain. This presentation allows a descriptive comparison of characteristics by pain status within and between clusters and does not imply that pain was used to define or generate the MRI-based clusters. Overall, no statistically significant differences were found between the groups for sex, working hours, chair choice, or working position (*p* > 0.05).

**Figure 1:**
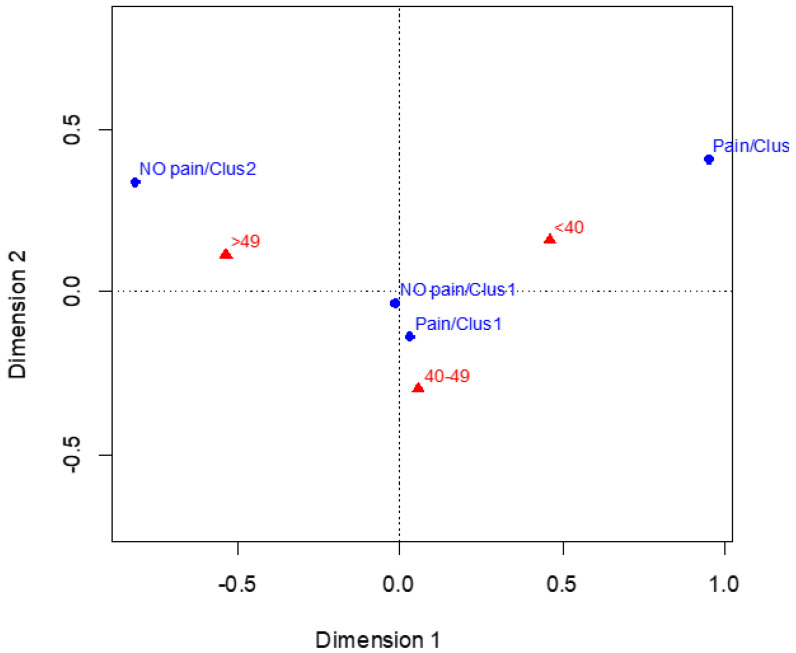
Correspondence analysis plot showing the relationship between age categories, MRI-based clusters, and neck pain status. Blue circles represent cluster–pain status combinations (Cluster 1 with pain, Cluster 1 without pain, Cluster 2 with pain, Cluster 2 without pain), while red triangles represent age categories (<40, 40–49, and >49 years). The spatial proximity between points reflects descriptive associations in the correspondence analysis space. Dimension 1 and Dimension 2 correspond to the first two dimensions of the correspondence analysis and represent the main sources of variability in the categorical data. This figure is intended for exploratory and descriptive purposes only and does not imply causal relationships.

Age distribution across MRI-based clusters and pain status is shown in Figure 1. Given the small number of participants in some subgroups, these findings should be interpreted with caution. Descriptively, younger participants (<40 years) were more frequently observed among those reporting pain despite belonging to the lower degenerative burden cluster, whereas older participants (>49 years) were more often represented among asymptomatic individuals within the same cluster. No formal interaction analyses were performed, and these observations should be considered exploratory.

This distribution suggests a nuanced, non-linear relationship between age, structural findings, and symptom reporting. Younger dentists demonstrate a higher prevalence of pain within the mild structural alteration profile, whereas older dentists show fewer symptoms within the same cluster. Meanwhile, the middle-aged group occupies an intermediate position, reflecting a more balanced distribution and a less defined clinical expression across profiles.

## 4. Discussion

The present exploratory study identified two cervical MRI-based structural profiles among actively practicing dentists, defined exclusively by differences in the overall burden and co-occurrence of degenerative MRI findings. Importantly, these profiles were derived solely from structural variables, and pain or disability measures were deliberately excluded from the clustering procedure to avoid circularity and overinterpretation. As a result, the identified profiles should be understood as descriptive representations of morphological variability rather than as clinical phenotypes. No individual MRI feature was independently associated with neck pain or disability, reinforcing the notion that structural abnormalities detected on MRI do not, by themselves, explain the clinical expression of neck pain in this occupational group. Although differences in pain prevalence and disability scores were observed between the identified profiles, these differences emerged only after cluster assignment and should therefore be interpreted as descriptive associations rather than evidence of a direct, causal, or predictive relationship. This interpretation is consistent with previous literature demonstrating a high prevalence of cervical degenerative changes in both symptomatic and asymptomatic individuals, which substantially limits the diagnostic and explanatory value of isolated imaging findings for pain attribution [10,13,14,15,16].

In line with earlier studies, individual MRI findings such as disc bulging, disc herniation, annular fissures, or Modic changes did not show consistent or robust associations with neck pain [9,11,13,14,15]. Although facet joint abnormalities and other osseous changes contributed to differentiating the identified structural profiles, these differences reflected variations in the cumulative degenerative burden rather than the presence of discrete pathological entities. Importantly, these structural distinctions did not translate into clear or proportional differences in symptom severity, further supporting the concept of a clinical–radiological mismatch in cervical spine disorders. Taken together, these findings underscore the importance of interpreting MRI findings within a broader clinical and occupational context rather than as direct indicators of symptom presence or intensity [9,10,11,15,16]. When degenerative changes were summarized at the individual level using a cumulative degenerative burden score, participants with neck pain tended to present a higher number of coexisting MRI abnormalities than those without pain. This finding supports the descriptive interpretation that a greater overall burden of structural changes, rather than any single MRI feature, may be associated with symptom reporting. However, substantial overlap between groups and the exploratory design preclude causal inference, and these observations should be interpreted as hypothesis-generating.

Age-related patterns emerged descriptively in the distribution of MRI-based profiles and symptom reporting. Younger dentists (<40 years) were more frequently observed among participants reporting pain despite belonging to the cluster characterized by a lower degenerative burden, whereas older dentists (>49 years) were more often represented among asymptomatic individuals within the same structural profile. The intermediate age group did not show a clear association with either cluster membership or pain status. These observations should be interpreted with caution, given the small size of some subgroups and the absence of formal interaction analyses. As such, the age-related findings should be considered hypothesis-generating rather than explanatory. One possible alternative explanation for this age-related pattern is the healthy worker effect. When applied to occupational settings, this concept suggests that individuals who remain active in physically demanding professions at older ages may represent a self-selected group of relatively healthier workers who have either adapted more effectively to occupational demands or are less susceptible to musculoskeletal symptoms. Consequently, older dentists in the present sample may not be representative of the broader population initially exposed to similar occupational risks, which may partially explain the lower symptom reporting despite comparable or greater structural degeneration. While these observations appear to contrast with traditional assumptions linking older age to higher pain prevalence [16], the present data do not allow conclusions regarding the underlying mechanisms.

No significant associations were observed between MRI-derived structural profiles and occupational characteristics such as working posture, weekly working hours, or chair support. Although ergonomic and biomechanical factors are widely recognized as contributors to neck pain among dental professionals [3,5,7,8], the absence of clear associations in this study may reflect methodological constraints. In particular, self-reported occupational measures may not adequately capture the complexity of biomechanical load, cumulative muscle fatigue, task variability, or contextual stressors inherent to dental practice. More objective assessments of posture, muscle activity, and workload exposure may therefore be required to better elucidate the relationship between occupational demands, structural changes, and symptom development [18,19,20]. Taken together, these findings reinforce the view that cervical MRI provides valuable information on structural characteristics but is insufficient to explain the multifactorial nature of neck pain among dentists. Rather than arising from isolated degenerative lesions, neck pain appears to reflect a complex interplay between structural burden, individual adaptation, occupational exposure, and neurophysiological modulation. Although a biopsychosocial framework is conceptually relevant for understanding neck pain, the present study was limited to structural and occupational factors, and no psychosocial variables were directly assessed. Consequently, any consideration of non-structural contributors to pain remains speculative and should be interpreted cautiously.

The identification of MRI-based structural profiles may nonetheless offer a useful descriptive framework for characterizing morphological heterogeneity within the dental profession. From a clinical and occupational health perspective, such profiling may help generate hypotheses for future research and inform the development of preventive strategies tailored to specific subgroups. However, given the exploratory design, the relatively small sample size, and the imbalance between clusters, these findings should not be interpreted as definitive phenotypes or used for individual-level clinical decision-making.

### Limitations

Several limitations should be acknowledged. First, pain was assessed in direct relation to professional activity, which may have introduced attribution bias by encouraging participants to link symptoms specifically to work-related exposure. Second, occupational and ergonomic variables were self-reported and therefore susceptible to recall bias. Third, although a biopsychosocial framework is conceptually relevant to neck pain, psychological and psychosocial factors were not measured in this study. The absence of psychosocial variables limits the ability to comprehensively assess non-structural contributors to pain and represents an important area for future research. In addition, the exploratory design, relatively small sample size, single-centre recruitment, and voluntary participation limit the generalizability of the findings and raise the possibility of selection bias. Accordingly, the results should be interpreted as hypothesis-generating and require confirmation in larger, longitudinal, and multicenter studies.

In addition, the ergonomic characterization of dental practice in this study was limited. Although basic occupational variables such as working posture, weekly working hours, and chair support were collected, several ergonomic factors known to influence cervical musculoskeletal load in dentistry were not assessed. These include patient–chair adjustment behaviours, sustained neck flexion combined with rotation, upper-back symptomatology, and the use of lumbar or forearm supports. Furthermore, psychosocial stressors and burnout, which are increasingly recognized as relevant in occupational musculoskeletal research, were not evaluated. The absence of these variables limits the ability to comprehensively interpret the contribution of occupational and ergonomic exposures to symptom expression.

## 5. Conclusions

Cervical MRI revealed a high prevalence of degenerative features among actively practicing dentists; however, no individual radiological finding showed a direct or independent association with the presence of neck pain. Using an exploratory hierarchical cluster analysis approach, two MRI-based structural profiles were identified, reflecting differences in the overall burden and co-occurrence of degenerative changes rather than discrete pathological entities. In this exploratory study, neck pain and higher disability scores were descriptively more frequent among participants belonging to the profile characterized by a higher degenerative burden. Importantly, these associations emerged from post hoc comparisons and were not used to define the clusters, and therefore should not be interpreted as explanatory, predictive, or causal. Age appeared to modulate symptom expression within structural profiles; however, these observations were based on small subgroup sizes and should be considered hypothesis-generating. Overall, the findings suggest that neck pain in dental professionals cannot be attributed to isolated MRI abnormalities, but rather reflects a complex interaction between structural characteristics, occupational exposure, and individual factors. Cervical MRI findings should therefore be interpreted within a broader clinical and occupational context, and caution is warranted when attributing symptoms solely to structural degeneration.

From a clinical and occupational health perspective, MRI-based structural profiling may provide a useful descriptive framework to support future research rather than immediate clinical decision-making. Given the exploratory design, limited sample size, and cross-sectional nature of the study, these results require confirmation in larger, longitudinal, and multicenter investigations that incorporate objective occupational assessments and psychosocial variables.

## Figures and Tables

**Table 1:** Demographic, professional, pain and magnetic resonance imaging (MRI) characteristics of all participants, and by subgroups of cervical pain.

Variables	All (*n* = 57)	With Pain (*n* = 40)	Without Pain (*n* = 17)	*p* Value
**Demographic**				
Sex Female	31 (54.4%)	23 (57.5%)	8 (47.1%)	0.469
Age				
<40	22 (38.6%)	16 (40.0%)	6 (35.3%)	
40-49	20 (35.1%)	14 (35.0%)	6 (35.3%)	0.925
>49	15 (26.3%)	10 (25.0%)	5 (29.4%)	
**Professional practices**				
Length of Service	18.3 (±12.2)	18.0 (±10.9)	18.7 (±14.9)	0.147
Weekly Hours > 36 h	28 (49.1%)	19 (47.5%)	9 (52.9%)	0.707
Chair Choice				0.884
*Without support*	22 (38.6%)	15 (37.5%)	7 (41.2%)	
*With 1 support*	19 (33.3%)	13 (32.5%)	6 (35.3%)	
*With 2 supports*	16 (28.1%)	12 (30.0%)	4 (23.5%)	
Working position				0.557
*Sitting*	21 (36.8%)	16 (40.0%)	5 (29.4%)	
*Standing*	4 (7.0%)	2 (5.0%)	2(11.8%)	
*Both*	32 (56.1%)	22 (55.0%)	10 (58.8%)	
**MRI measures**				
Fissure in the annulus fibrosus	6 (10.5%)	4 (10.0%)	2 (11.8%)	0.843
Bulging disc	38 (66.7%)	25 (62.5%)	13 (76.5%)	0.379
Herniated disc	31 (54.4%)	22 (55.0%)	9 (52.9%)	0.886
Canal stenosis	15 (26.3%)	11 (27.5%)	4 (23.5%)	0.755
Presence of osteophytes	20 (35.1%)	13 (32.5%)	7 (41.2%)	0.530
Facet abnormalities	17 (29.8%)	13 (32.5%)	4 (23.5%)	0.498
**Pain measures**				
NDI score 15>	30 (52.6%)	26 (65.0%)	4 (23.5%)	0.004

**Table 2:** Characteristics of MRI-based clusters.

MRI Measures	Cluster 1 (*n* = 40)	Cluster 2 (*n* = 17)	*p* Value
Fissure in the annulus fibrosus	4 (10.0%)	2 (11.8%)	0.843
Bulging disc	25 (65.8%)	13 (76.5%)	0.306
Herniated disc	22 (55.0%)	9 (52.9%)	0.886
Canal stenosis	11 (27.5%)	4 (23.5%)	0.755
Presence of osteophytes	13 (32.5%)	7 (41.2%)	0.530
Facet abnormalities	13 (32.5%)	4 (23.5%)	0.498
**Neck Pain measures**			
Pain	7(17.5%)	3 (17.6%)	0.001
NDI > 20	26 (65.0%)	4 (23.5%)	0.004

**Table 3:** Participants’ characteristics related to MRI-based clusters with and without pain.

	Cluster 1 (*n* = 40)		Cluster 2 (*n* = 17)		
**Demographic**	Without pain	With pain	Without pain	With pain	*p* Value
Sex Female	6 (42.9%)	2 (66.7%)	20 (60.6%)	3 (42.9%)	0.609
Age					0.432
<40	5 (35.7%)	1 (33.3%)	11 (33.3%)	5 (71.4%)	
40-49	5 (35.7%)	1 (33.3%)	12 (36.4%)	2 (28.6%)	
>49	4 (28.6%)	1 (33.3%)	10 (30.3%)	0 (0.0%)	
**Professionals**					
Weekly Hours > 36 h	7 (50.0%)	2 (66.7%)	16 (48.5%)	5 (71.4%)	0.921
Chair Choice					0.989
*Without support*	6 (42.9%)	1 (33.3%)	13 (39.4%)	2 (28.6%)	
*With 1 support*	5 (35.7%)	1 (33.3%)	10 (30.3%)	3 (42.9%)	
*With 2 supports*	3 (21.4%)	1 (33.3%)	10 (30.3%)	2 (28.6%)	
Working position					0.223
*Sitting*	3 (21.4%)	2 (66.7%)	14(42.4%)	2 (28.6%)	
*Standing*	1 (7.1%)	1 (33.3%)	2 (6.1%)	0 (0%)	
*Both*	10(71.4%)	0 (0%)	33 (100%)	7 (100%)	

## Data Availability

The data supporting the findings of this study are available from the corresponding author upon reasonable request. Due to privacy and ethical restrictions, the dataset is not publicly available.

## Abbreviations

The following abbreviations are used in this manuscript:

MRI	Magnetic Resonance Imaging
NDI	Neck Disability Index
NP	Neck Pain
SD	Standard Deviation

## References

[1] Wu A.-M., Cross M., Elliott J.M., Culbreth G.T., Haile L.M., Steinmetz J.D., Hagins H., A Kopec J., Brooks P.M., Woolf A.D. (2024). Global, Regional, and National Burden of Neck Pain, 1990–2020, and Projections to 2050: A Systematic Analysis of the Global Burden of Disease Study 2021. Lancet Rheumatol..

[2] Wu H., Li Y., Zou C., Guo W., Han F., Huang G., Sun L. (2025). Global Burden of Neck Pain and Its Gender and Regional Inequalities from 1990–2021: A Comprehensive Analysis from the Global Burden of Disease Study 2021. BMC Musculoskelet. Disord..

[3] AlOtaibi F., Nayfeh F.M.M., Alhussein J.I., Alturki N.A., Alfawzan A.A. (2022). Evidence Based Analysis on Neck and Low Back Pain among Dental Practitioners—A Systematic Review. J. Pharm. Bioallied Sci..

[4] Thorat N.C., Sahana S., Chauhan N., Singh T.P., Khare A. (2022). Prevalence of Musculoskeletal Pain in Dentists; A Systematic Review and Meta-Analysis. J. Head Neck Physicians Surg..

[5] Rafeemanesh E., Omidi-Kashani F., Chamani A., Allahdad S. (2025). Occupational and Non-Occupational Risk Factors for Neck Pain in Dentists: A Systematic Review and Meta-Analysis. Arch. Bone Jt. Surg..

[6] Kawtharani A.A., Chemeisani A., Salman F., Haj Younes A., Msheik A. (2023). Neck and Musculoskeletal Pain Among Dentists: A Review of the Literature. Cureus.

[7] Hodačová L., Pilbauerová N., Čermáková E., Machač S., Schmidt J., Hodač J., Kapitán M. (2022). The Prevalence and Development of Neck and Lower Back Pain and Associated Factors in Dentistry Students—A Long-Term Prospective Study. Int. J. Environ. Res. Public Health.

[8] Park H.-S., Kim J., Roh H.-L., Namkoong S. (2015). Analysis of the Risk Factors of Musculoskeletal Disease Among Dentists Induced by Work Posture. J. Phys. Ther. Sci..

[9] Peng B., DePalma M.J. (2018). Cervical Disc Degeneration and Neck Pain. J. Pain Res..

[10] Farrell S.F., Smith A.D., Hancock M.J., Webb A.L., Sterling M. (2019). Cervical Spine Findings on MRI in People with Neck Pain Compared with Pain-Free Controls: A Systematic Review and Meta-Analysis. J. Magn. Reson. Imaging.

[11] Yang X., Karis D.S.A., Vleggeert-Lankamp C.L.A. (2020). Association between Modic Changes, Disc Degeneration, and Neck Pain in the Cervical Spine: A Systematic Review of Literature. Spine J..

[12] Okada E., Matsumoto M., Fujiwara H., Toyama Y. (2011). Disc Degeneration of Cervical Spine on MRI in Patients with Lumbar Disc Herniation: Comparison Study with Asymptomatic Volunteers. Eur. Spine J..

[13] Jensen R.K., Dissing K.B., Jensen T.S., Clausen S.H., Arnbak B. (2023). The Association between Cervical Degenerative MRI Findings and Self-Reported Neck Pain, Disability and Headache: A Cross-Sectional Exploratory Study. Chiropr. Man. Ther..

[14] Hill L., Aboud D., Elliott J., Magnussen J., Sterling M., Steffens D., Hancock M.J. (2018). Do Findings Identified on Magnetic Resonance Imaging Predict Future Neck Pain? A Systematic Review. Spine J..

[15] Rudy I.S., Poulos A., Owen L., Batters A., Kieliszek K., Willox J., Jenkins H. (2015). The Correlation of Radiographic Findings and Patient Symptomatology in Cervical Degenerative Joint Disease: A Cross-Sectional Study. Chiropr. Man. Ther..

[16] Siivola S.M., Levoska S., Tervonen O., Ilkko E., Vanharanta H., Keinänen-Kiukaanniemi S. (2002). MRI Changes of Cervical Spine in Asymptomatic and Symptomatic Young Adults. Eur. Spine J..

[17] Al Shammari G.A.A., Al-Esawi S.R., Taher A., Albujeer A.H. (2020). Magnetic Resonance Imaging (MRI) Findings of Cervical Spine Derangement (CSD) Among Iraqi Working Dentists. Open Dent. J..

[18] Horga L.M., Hirschmann A.C., Henckel J., Fotiadou F., Di Laura A., Torlasco C., D’Silva A., Sharma S., Moon J.C., Hart A.J. (2020). Prevalence of Abnormal Findings in 230 Knees of Asymptomatic Adults Using 3.0 T MRI. Skeletal Radiol..

[19] Katzman G.L., Dagher A.P., Patronas N.J. (1999). Incidental Findings on Brain Magnetic Resonance Imaging from 1000 Asymptomatic Volunteers. JAMA.

[20] Dünschede J., Ruschil C., Bender B., Mengel A., Lindig T., Ziemann U., Kowarik M.C. (2023). Clinical-Radiological Mismatch in Multiple Sclerosis Patients During Acute Relapse: Discrepancy Between Clinical Symptoms and Active, Topographically Fitting MRI Lesions. J. Clin. Med..

[21] Mohammadi T., D’Ascenzo F., Pepe M., Bonsignore Zanghì S., Bernardi M., Spadafora L., Frati G., Peruzzi M., De Ferrari G.M., Biondi-Zoccai G. (2023). Unsupervised Machine Learning with Cluster Analysis in Patients Discharged After an Acute Coronary Syndrome: Insights from a 23,270-Patient Study. Am. J. Cardiol..

[22] Paoletti M., Camiciottoli G., Meoni E., Bigazzi F., Cestelli L., Pistolesi M., Marchesi C. (2009). Explorative Data Analysis Techniques and Unsupervised Clustering Methods to Support Clinical Assessment of Chronic Obstructive Pulmonary Disease (COPD) Phenotypes. J. Biomed. Inform..

[23] Collins S.L., Moore R.A., McQuay H.J. (1997). The Visual Analogue Pain Intensity Scale: What Is Moderate Pain in Millimetres?. PAIN.

[24] Jorritsma W., Dijkstra P.U., de Vries G.E., Geertzen J.H.B., Reneman M.F. (2012). Detecting Relevant Changes and Responsiveness of Neck Pain and Disability Scale and Neck Disability Index. Eur. Spine J..

[25] Kato S., Takeshita K., Matsudaira K., Tonosu J., Hara N., Chikuda H. (2012). Normative Score and Cut-off Value of the Neck Disability Index. J. Orthop. Sci..

